# Neuromyelitis Optica Spectrum Disorder With Unilateral Retrobulbar Neuritis: A Case Report

**DOI:** 10.7759/cureus.75410

**Published:** 2024-12-09

**Authors:** Emran Lyutfi, Eva Kasabova, Boyko Matev, Kaloyana Shangova, Ara Kaprelyan

**Affiliations:** 1 First Clinic of Neurology, University Hospital “St. Marina”, Varna, BGR; 2 Neurology and Neurosciences, Medical University of Varna, Varna, BGR; 3 Diagnostic Imaging, University Hospital “St. Marina”, Varna, BGR; 4 Imaging Diagnostics, Interventional Radiology and Radiotherapy, Medical University of Varna, Varna, BGR; 5 Ophthalmology and Visual Sciences, Medical University of Varna, Varna, BGR; 6 Neurology and Neuroscience, Medical University of Varna, Varna, BGR

**Keywords:** aquaporin 4 (nmo-igg) antibodies, neuromyelitis optica spectrum disorder (nmosd), ophthalmoplegia syndrome, recurrent painful ophthalmoplegia, short-lasting unilateral neuralgiform headache attacks with conjunctival injection and tearing (sunct), transverse myelitis, unilateral optic neuritis

## Abstract

Neuromyelitis optica spectrum disorder (NMOSD) includes conditions with autoimmune genesis, which are manifested by attacks of optic neuritis (ON) and transverse myelitis (TM), and also express aquaporin 4 (NMO-IgG) or myelin oligo-endocytic glycoprotein (MOGAb) antibodies. In rare cases, the disease may also have a clinical presentation with only TM, without ON or with ON, without TM. These conditions are also included in the spectrum. We present a case of a 50-year-old patient with complaints of blurry vision in the left eye, neck pain, and numbness of the hands a few months prior to hospitalization. After a consultation with an ophthalmologist, magnetic resonance imaging (MRI) of the brain was performed, and findings showed left ON. Serological findings showed antibodies for aquaporin 4 with a ratio of 1:100, which is a pathognomic marker for NMOSD. Upon admission to the clinic, the complaints persisted. The neurological status revealed decreased vision of the left eye, pain in the Valleix points in the cervical region, and hyperesthesia including dermatomes C5-C8, more prominent for the right arm. Routine blood tests showed leukocytosis, neutrophilia, and lymphocytosis. Additional virology studies were performed to rule out neuroborreliosis, neurosyphilis, and human immunodeficiency virus (HIV). MRI of the cervical spine revealed dural sac compression from degenerative spinal disease. Electromyography (EMG) showed radiculopathy at the C5-C8 levels and neuropathy of n. ulnaris sinistra and n. medianus dextra. Consultation with an ophthalmologist revealed a decrease in the left eye visus. The treatment plan consisted of methylprednisolone 1000 mg for five days and gastroprotection with famotidine 40 mh p.o. Further consultations with the ophthalmologist were done in the following days to monitor the visual status of the patient. After completion of the diagnostic and treatment plan, slight recovery of the visus and reversal of the pain symptomatology was reported. Based on the history, clinical findings, ophthalmologic examination, brain and neck imaging (MRI), and laboratory results (NMO-IgG ratio 1:100 ), NMOSD with left retrobulbar neuritis (Devic's disease) was concluded as the final diagnosis. Multiple sclerosis (MS; retrobulbar neuritis) first attack, systemic connective tissue disease (systemic lupus erythematosus, Sjögren's syndrome, and antiphospholipid syndrome), and neuro-infection were discussed. Clinical suspicion of intracranial tumors was ruled out. Corticosteroids and gastroprotectors were prescribed for oral use under the supervision of a neurologist. Regular check-ups were performed. The reported case of NMOSD with unilateral optic nerve involvement is a rare condition in neurological practice. It requires an individualized approach to diagnose and apply treatment. Patient follow-up is a key starting point toward gathering more information about autoimmune processes in the central nervous system (CNS) and toward finding faster, more reliable, and cheaper clinical and instrumental methods of diagnosis, which in turn may lead to more timely initiation of treatment, proportionally leading to improvement in the patient's quality of life.

## Introduction

Definition and history

The term neuromyelitis optica spectrum disorder (NMOSD) includes conditions with an autoimmune genesis that are manifested by attacks of optic neuritis (ON) and transverse myelitis (TM) and also express aquaporin 4 (NMO-IgG) antibodies. In rare cases, the disease may also have a clinical presentation with only TM without ON or with ON without TM. These conditions are also included in the spectrum [1].

Opticomyelitis was first described by Eugene Devic in 1894. The name neuromyelitis optica (Devic’s disease) was given later by Gault, who was Devic’s student [2].

The discovery of opticomyelitis-specific antibodies (NMO-IgG) by Vanda Lennon in 2004 led to improvement in the early diagnosis and differential diagnosis of the disease. Recent studies show that in rare cases, patients with NMOSD could be seronegative, and specific myelin oligo-endocytic glycoprotein (MOGAb) antibodies were present. In the past, these antibodies have been detected in patients with acute disseminated encephalomyelitis [1].

Etiology and epidemiology

The etiology of NMOSD is still unclear. The most common theories suggest genetic origins. In previous classifications, NMOSD was considered a type of MS. According to recent studies, genetically, NMOSD is more similar to systemic lupus erythematosus (SLE) than to MS [3].

The incidence and prevalence of NMOSD vary in studies from different regions of the world (between 0.28-0.73/100,000 and 0.52-10/100,000), but the condition is most prevalent in Asia. The disease usually debuts between the ages of 20 and 40 years; however, the first attack can occur in children and adults alike. The female-to-male ratio is the highest in Japan at 10:1 [4].

Clinical presentation

Clinically, the disease is characterized by two main components: 1) severe episodes of ON leading to complete loss of vision and in most cases incomplete recovery [5] and 2) TM, manifested by severe paraparesis, bladder and bowel dysfunction, and headache or other pain syndromes [6].

There are rare cases where brain stem dysfunction is also involved and clinically patients experience severe vomiting, hiccups, disturbed sleep, and impaired secretion of antidiuretic hormone [7-8].

Diagnosis

Diagnosis is based on the basic clinical features and is supported by further investigations. The main diagnostic marker is the detection of NMO-IgG (AQP4 Ab) and MOGAb in serum. In 2015, international consensus diagnostic criteria were established to separate NMOAD into AQP4-IgG status (with testing, without testing, or unknown) and to define the clinical and magnetic resonance imaging (MRI) features of NMOAD [9].

Along with serological analysis, MRI is one of the main diagnosis requirements. Longitudinal lesions of the myelon, occupying more than half of its axial width, and with an involvement of three or more segments are found. Subsequent studies have shown that lesions can also develop in the cerebellum as the disease evolves, and these were included in the 2015 revised criteria. In recent years, optical coherence tomography (OCT) has been widely used in the diagnosis and follow-up of neuroimmune diseases. In patients with opticomyelitis, OCT detects severe damage in the early stages of ON. It leads to visual impairment, which on the other hand correlates with a negative impact on the quality of life. Pathological results are reported in both AQP4 Ab- and MOG Ab-positive patients. The place of OCT as a method to aid in differential diagnosis, therapeutic decision-making, and patient follow-up has yet to be determined in future studies [10-12].

Treatment

NMOSD therapy includes four main directions: 1) treatment of the attacks, 2) immunosuppressive treatment, 3) symptomatic treatment, and 4) physiotherapy and rehabilitation.

Due to the low incidence of the disease, severe attacks, early possibility of disability, and lethality in untreated patients, there is a lack of data with a high degree of reliability on the therapeutic strategies. The attacks are treated with pulse therapy with the corticosteroid methylprednisolone 1 g/day for five days, followed by switching to oral prednisone therapy 1 mg/kg/day for up to six to 12 months. The dose of methylprednisolone may be increased to two grams daily for patients who do not show improvement.

Due to the low incidence of the disease, severe attacks, early disabling potential, and lethal outcomes in untreated patients, a high degree of reliability data on therapeutic strategies is lacking. The attacks are treated with pulse therapy with the corticosteroid methylprednisolone 1 g/day in a five-day course, followed by switching to peroral therapy with corticosteroid 1 mg/kg/day for six to 12 months with step-by-step dose reduction. The dose of methylprednisolone may be increased to two grams daily for patients who do not respond well to treatment.

In instances where a high dose of corticosteroids has yielded an inadequate response, plasmapheresis is administered as a therapeutic measure. Plasmapheresis is the preferred option for patients presenting with severe neurological deficits, such as those observed in TM, or in cases where corticosteroid treatment has been ineffective in previous attacks. The lack of serious side effects or complications associated with plasmapheresis makes it an ideal first-line therapy for the treatment of attacks. Additionally, studies have demonstrated that early administration of plasmapheresis is associated with a more favorable outcome.

Immunosuppressive therapy is used for attack prophylaxis. The most commonly administered immunosuppressive agents are prednisone, azathioprine, mitoxantrone, rituximab, mycophenolate, methotrexate, cyclophosphamide, and immunoglobulins. Plasmapheresis is also a frequently utilized method of treatment. The selection of a specific medication is predominantly determined by the availability of the medication, the potential adverse effects, and the experience of the treating physician with the medication in question. The most common approach was to commence treatment following an attack with oral corticosteroids, introduce the selected immunosuppressant, and gradually withdraw the corticosteroid. It is of significant importance to provide symptomatic treatment for pain, stiffness, pelvic reservoir disorders, and fatigue. It is of particular importance to provide rehabilitation for patients with opticomyelitis, as even the initial episode of the disease can result in significant disability [13].

## Case presentation

**Figure 1:**
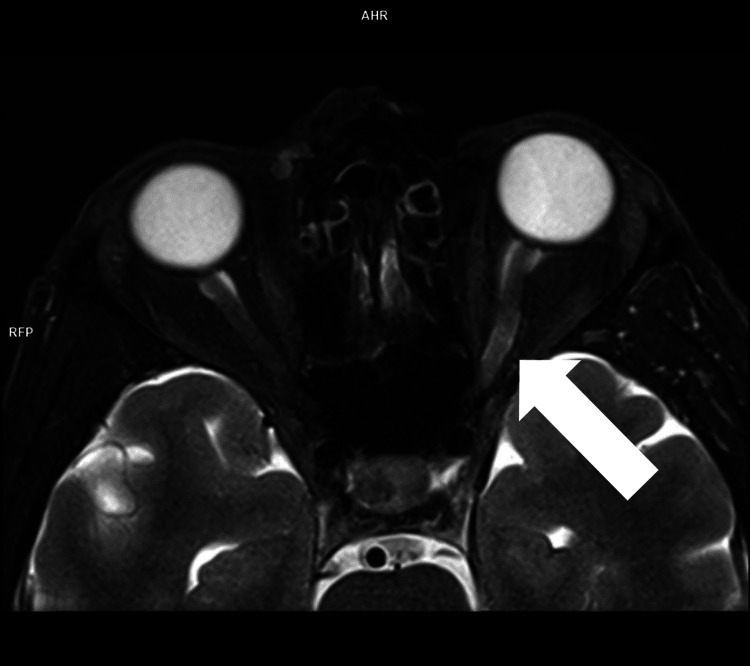
Magnetic resonance imaging (MRI head): transverse T2-weighted sequence White arrow: hyperintensity of the left optic nerve on the transverse T2 Dixon sequence water-only imaging.

**Figure 2:**
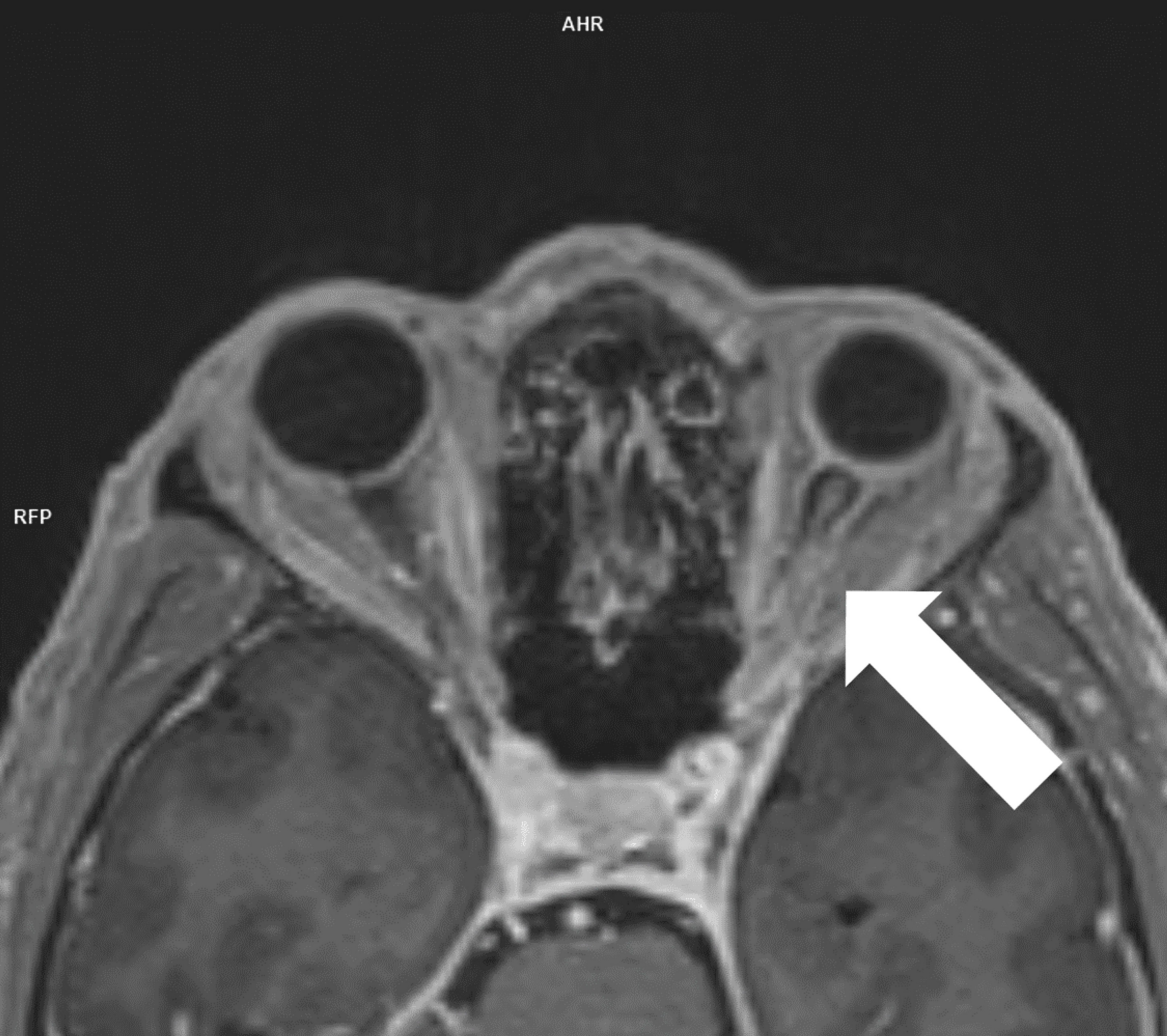
MRI head: transverse contrast-enhanced T1-weighted sequence. White arrow: Patchy enhancement of the left optic nerve on the transverse T1-weighted contrast-enhanced imaging.

We present a case of a 50-year-old patient admitted to the First Clinic of Neurology, University Hospital of "St. Marina," Varna, for blurry vision of the left eye, neck pain, and numbness of the hands a few months prior to hospitalization. A few weeks ago, an MRI of the brain was performed at the suggestion of an ophthalmologist. The findings of the hyperintensity of the left optic nerve on axial T2-weighted imaging and the contrast enhancement of the structure on coronal fat-suppressed T1-weighted imaging were indicative of ON (Figures 1-2).

The patient was advised to visit a neurologist, who suggested performing a serology lab test for NMO-IgG (AQP4 Ab). Antibodies for aquaporin 4 were positive at a ratio of 1:100, which is a pathognomic marker for NMOSD. After these findings, the patient was planned for admission to perform the remaining tests. The neurological status revealed blurry and decreased vision of the left eye, pain in the Valleix points in the cervical region, and hyperesthesia including dermatomes C5-C8, more prominent for the right arm. The blood tests showed leucocytosis, neutrophilia, and lymphocytosis. There was no deviation from the remaining routine laboratory tests (Table 1).

**Table 1:** Blood test The bold text indicates the abnormal results.

Blood test parameters	Laboratory reference ranges in healthy males	Results
C-reactive protein- mg/L	0-5	1.2
Neutrophils# - 10*9/L	1.78-7	8.93
Neutrophils - %	39-77	67.1
Alanine transaminase (ALAT) - serum - U/L	10-49	40
Aspartate aminotransferase (ASAT)- serum -U/L	0-34	26
Basophils # - 10*9/L	0-0.2	0.05
Basophils - %	0-1.75	0.4
Glucose - mmol/L	4.1-5.9	5
Eosinophils # - 10*9/L	0.03-0.47	0.05
Eosinophils - %	0.5-5.5	0.4
Red blood cells (RBC)- 10*12/L	4.57-5.98	5.41
K+ -mmol/L	3.5-5.5	4.47
Creatinine- serum -mmol/L	62-115	73
White blood cells (WBC)- 10*9/L	3.79-10.33	13.29
Lymphocytes#	1.07-3.12	3.53
Lymphocytes %	20-44	26.6
Monocytes #	0.24-0.73	0.73
Monocytes %	1.5-9.5	5.5
Na+ - mmol/L	132-146	140
International normalised ratio (INR)	0.9-1.15	1.04
Triglycerides	0-1.7	0.93
Platelet count (PLT)- 10*9/L	140-440	382
Urea - mmol/L	3.2-8.2	5.88
Hematocrit (HCT) - L/L	0.395-0.505	0.483
Hemoglobin (HGB) - g/L	135-172	159
Cl- - mmol/L	99-109	104.5

**Figure 3:**
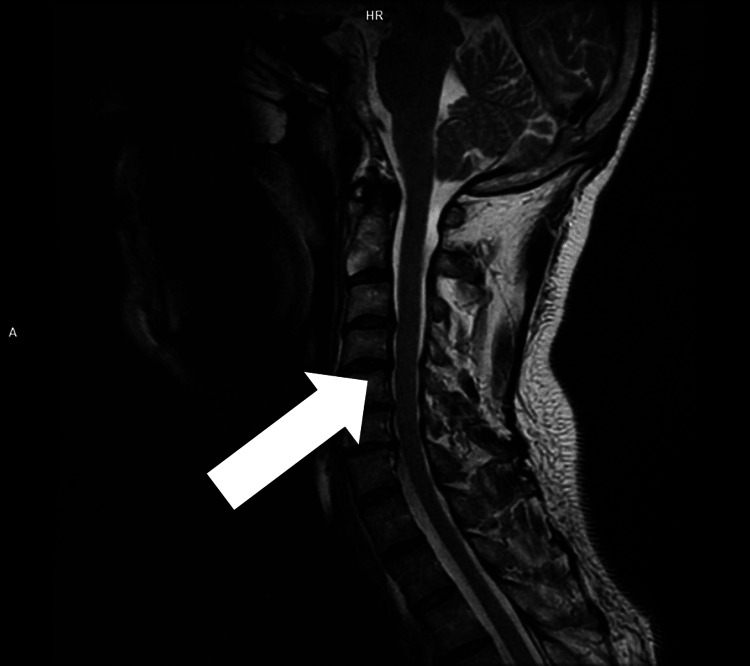
MRI of the cervical spine White arrow: degenerative cervical disc disease on sagittal T2-weighted imaging

Additional virology studies were performed with the patient's consent to rule out neuroborreliosis, neurosyphilis, and HIV. Immunological investigations were also performed, the results of which were not available during the stay of the patient. An MRI of the cervical spine revealed dural sac compression from degenerative spinal disease (Figure 3). Electromyography (EMG) showed radiculopathy at the C5-C8 levels and neuropathy at n. ulnaris sinistra and n. medianus dextra.

Consultation with an ophthalmologist was administered and revealed a pathological decrease in the left vision. Additional conjunctivitis was reported. The treatment plan consisted of methylprednisolone 1000 mg for five days and gastroprotection with famotidine 40 mg p.o. Further consultations with the ophthalmologist were done in the following days to monitor the visual status of the patient. After completion of the diagnostic and treatment plan, slight recovery of the visus and reversal of the pain symptomatology was reported.

Based on the history, clinical findings, ophthalmologic examination, brain and neck imaging (MRI), and laboratory results (seropositive for anti-aquaporin 4 antibody), NMOSD with left retrobulbar neuritis (Devic's disease) was concluded as the final diagnosis. MS (retrobulbar neuritis) first attack, systemic connective tissue disease (systemic lupus erythematosus, Sjögren's syndrome, and antiphospholipid syndrome), and neuro-infection were discussed. Clinical suspicion of intracranial tumors was ruled out.

Corticosteroids and gastroprotectors were prescribed for oral use under the supervision of a neurologist.

On follow-up examination, the results of immunologic testing for rheumatoid diseases showed elevated rheumatoid arthritis antibodies (ELISA-ANTI-CCP) 359.8 with normal to 20 and positive antinuclear bodies for ANA and ANF. He was referred to a rheumatologist for diagnostic clarification and follow-up.

## Discussion

Opticomyelitis is an autoimmune disease manifested by attacks of ON and TM. It affects people at a young age. Clinically, it is characterized by severe episodes of ON and/or in combination with transversal myelitis, manifested by severe paraparesis, bladder and bowel dysfunction, and headache or other pain syndromes [14].

Recovery after an attack is usually incomplete, which is why early and accurate diagnosis and proper treatment are important [13].

The discovery of the opticomyelitis-specific antibody aquaporin 4 (NMO-IgG) led to early diagnosis of the disease, which on the other hand expanded the possibility for an early differential diagnosis [15].

The differential diagnosis of the disease is very broad and includes several diseases that can be classified into several groups [1]:

Non-infectious inflammatory diseases

This group is represented by relapsing and progressive MS, Behcet's disease, neurosarcoidosis, rheumatic diseases (such as systemic lupus erythematosus, Sjögren’s syndrome, overlap syndromes, primary CNS vasculitis, systemic vasculitis), anti-NMDA receptor encephalitis, among others [16].

The abovementioned Behcet’s disease finds a particular place in differential diagnosis because 3% of the cases could have only neurological manifestations (neuro-Behcet) with headache, hemiparesis, lesions of the cranial nerves, sensory involvement, and other complications [17].

Infectious diseases

This vast group of infectious diseases involves the CNS: viral myelitis, which can be caused by varicella-zoster virus, herpes simplex virus, HIV, HTLV 1/2, tick-borne encephalitis virus, enteroviruses, Epstein-Barr virus, cytomegalovirus, West Nile virus, and poliovirus, neurotuberculosis, neuro-borreliosis, *Bartonella henselae*, neurosyphilis, and other rare infections.

Vascular diseases

These conditions are related to blood vessel damage. The conditions are rare but lead to major disability. They include stroke or spinal cord infarction and sinus thrombosis (bilateral papilledema).

Neoplastic diseases

Rare cases are reported with CNS lymphoma involvement or intramedullary tumors (e.g., ependymoma and astrocytoma, hemangioblastoma, rarely others).

Genetic and metabolic diseases

This group is presented by vitamin B12, vitamin E, copper or biotinidase deficiencies, Leber's hereditary optic neuropathy, and leukodystrophies (including Alexander's disease) [1].

There are various clinical cases of vitamin b12 deficiency and chronic anemia that have similar clinical presentation and mimic Devic’s disease due to the destruction of myelin of the axons and leading to myelopathy with metabolic etiology [18].

Other rare diseases 

Other rare diseases may have similar clinical features: idiopathic intracranial hypertension (bilateral papilledema); traumatic spinal cord, brain, brainstem, or optic nerve damage; and compressive myelopathy [1].

Other conditions that might mimic opticomyelitis with clinical features such as headache and cranial nerve involvement are short-lasting unilateral neuralgiform headache attacks with conjunctival injection and tearing (SUNCT) and recurrent painful ophthalmoplegia or ophthalmoplegia syndrome. SUNCT includes severe unilateral headache, associated with painfully decreased eye movements on the same side of the head. It is a rare condition from the group of primary headaches [19].

Recurrent painful ophthalmoplegia includes severe unilateral headache, associated with painfully decreased eye movements on the same side of the head. There is a reported case that presents a similarity between the abovementioned condition and painful ophthalmoplegia [20].

Based on the medical history of the patient, i.e., a 50-year-old male with symptoms of blurry and decreased vision of the left eye, neck pain, and numbness of the upper limbs a few months prior to the hospitalization; neurological status, i.e., decreased vision of the left eye, pain in the Valleix points in the cervical region, and paresthesia including dermatomes C5-C8, more prominent for the right arm; and the lab results, i.e., with positive NMO-IgG (AQP4 Ab) and MRI findings of retrobulbar neuritis in left, NMOSD with left retrobulbar neuritis (Devic's disease) was concluded as the final diagnosis.

The routine laboratory findings showed an active inflammation - leucocytosis, neutrophilia, and lymphocytosis. Additional virology studies were performed with the patient's consent to rule out neuroborreliosis, neurosyphilis, and neurologic complications from HIV. Immunological investigations were also performed, the results of which were not available during the stay of the patient. However, on follow-up examination, the results of immunologic testing for rheumatoid diseases showed elevated rheumatoid arthritis antibodies (ELISA-ANTI-CCP) 359.8 with normal to 20 and positive antinuclear bodies for ANA and ANF. He was referred to a rheumatologist for diagnostic clarification and follow-up.

MS (retrobulbar neuritis) first attack, systemic connective tissue disease (systemic lupus erythematosus, Sjögren's syndrome, antiphospholipid syndrome), and neuro-infection were discussed as potential differential diagnoses. Clinical suspicions of intracranial tumors were ruled out.

## Conclusions

The reported case of NMOSD with unilateral optic nerve involvement is a rare condition in neurological practice. It requires an individualized approach to diagnose and apply treatment. Patient follow-up is a key starting point toward gathering more information about autoimmune processes in the CNS and toward finding faster, more reliable, and cheaper clinical and instrumental methods of diagnosis, which in turn may lead to more timely initiation of treatment and proportionally lead to improvement the patient's quality of life.
